# NAV3 Is a Novel Prognostic Biomarker Affecting the Immune Status of the Tumor Microenvironment in Colorectal Cancer

**DOI:** 10.1155/2022/8337048

**Published:** 2022-06-30

**Authors:** Menglong Li, Zhiqiang Wang, Shuai Dong, Yang Xu

**Affiliations:** Department of Colorectal Surgery, The Second Hospital of Tianjin Medical University, Tianjin, China

## Abstract

Colorectal cancer (CRC) is one of the most common malignant tumors in the world. Tumor microenvironment (TME) plays a crucial role in the development of CRC. With the deep understanding of TME function, growing studies have demonstrated that alteration in tumor-infiltrating immune cells (TICs) and gene expressions are associated with clinical outcomes of various tumors. In this study, we aimed to recognize critical prognostic genes involved in immune states in TME of CRC. Hence, the proportion of TICs and the number of immune and stromal components in CRC samples from TCGA datasets were calculated by the use of CIBERSORT and ESTIMATE calculation methods. Different assays were applied to collect differential expression genes (DEGs) shared by the ImmuneScore and StromalScore. DEGs were further analyzed by the use of univariate Cox regression. Our attention focused on neuron navigator 3 (NAV3) which was highly expressed in CRC specimens and associated with an advanced clinical stage and poor prognosis of CRC patients. KEGG assays revealed that NAV3 may be involved in Alzheimer disease, amyotrophic lateral sclerosis, Huntington disease, FoxO signaling pathway, and human papillomavirus infection. Correlation assays showed that macrophage M0 and B cells memory, NK cells activated, dendritic cells resting, T cells CD4 memory activated, and T cells CD8 were correlated with NAV3 expression, indicating that NAV3 may represent the immune status of TME. Finally, RT-PCR confirmed that NAV3 expression was distinctly increased in CRC cells, and its knockdown suppressed the proliferation of CRC cells. Overall, NAV3 could be used as a novel predictor for TME of CRC and might be a novel prognostic biomarker. In the future, drugs targeting NAV3 might be developed as a potential immunotherapy for CRC patients.

## 1. Introduction

Colorectal cancer (CRC) refers to the third most commonly detected cancer, taking up ~10% of death caused by cancer and having been recognized to be the 2nd cause of death arising from cancer in the whole world [1, 2]. Over 1 million people have CRC each year. Moreover, the death rate from certain diseases is close to 33 percent among developed nations [3]. The main reason for the death of patients with colorectal carcinoma is liver metastasis [4]. The overall survival of patients has not significantly improved thus far though the diagnosing and treating strategy for liver metastasis of CRC has been significantly optimized over the past few years [5, 6]. Thus, new markers in terms of CRC should be searched, so the biological characteristic exhibited by tumors is able to be accurately identified, and clinical treatment can be facilitated.

The tumor microenvironment (TME) components within solid tumors, in particular chemokines, arouse huge interest of the academic world [7]. More and more studies have confirmed several specific functions of TME cells (e.g., B cells memory, macrophages M2, and eosinophils) in tumor development and cancer-related cachexia, via the modulation of different procachectic elements [8, 9]. It has been confirmed that TME may promote cancer development via influencing the immune surveillances and weaken the abilities of chemotherapy targeting tumor cells [10, 11]. Recently, in lung adenocarcinoma, TME-associated ImmuneScore was confirmed to be a novel predictor for tumor metastasis, relapses, and clinical outcome [12]. Furthermore, more and more studies have provided evidences that the ImmuneScore can be used to classify lung cancer [13, 14]. However, the effects of TME in CRC remained largely unclear.

The development of TCGA has allowed us to identify immune-related biomarkers for CRC on a large scale. In this study, several immune-related biomarkers were reported, related to the CRC patient's prognosis. Neuron navigator 3 (NAV3) pertains to the family of neuron navigator containing 3 members [15]. It has been confirmed that this gene family is involved in several cellular processes, such as cell migration, microtubule dynamics, membrane fusion, regulation of gene expression, signal transduction, and protein degradation [16, 17]. To date, only a few studies have performed on NAV3 in cancer, particularly its relationship with the immune responses in CRC. For instance, NAV3 was identified as a potential target of miR-21-3p and its knockdown increases resistance [18]. The data in this research suggested that NAV3 could function as a novel prognosis biological marker involved in the immune responses.

## 2. Materials and Methods

### 2.1. Data Sources and Processing

The CBioPortal (http://www.cbioportal.org) was applied to obtain gene mRNA expression information and relevant clinical data relating to the CRC case within TCGA projects. The Illumina HiSeq 2000 was applied to produce the mRNA-Seq data from TCGA. In this study, 460 CRC samples and 41 nontumor samples were collected for further study.

### 2.2. Generation of ESTIMATEScore, StromalScore, and ImmuneScore

An algorithm called ESTIMATE has been designed. In this algorithm, the expression characteristics of specific genes in immune cells and stromal cells are analyzed to calculate immune and stromal scores for the prediction of the invasion of nontumor cells [19]. The ESTIMATE algorithm was applied for the determination of immune and stromal cells' proportion within the TME with the use of R. Kaplan-Meier assays were applied to examine the clinical outcome. Log-rank *p* < 0.05 had statistical significance.

### 2.3. Recognition of TME-Associated DEGs

We screened DEGs of the low StromalScore group and high StromalScore group and the low ImmuneScore group and high ImmuneScore group. The differential expression assays were performed based on the “limma” R package. The standards in terms of DEG identification referred to false discovery rate- (FDR-) regulated *p* < 0.05 and |log2(foldchange)| > 1. We drew the identified genes within the thermal map applying the “ggplot2” R package.

### 2.4. Functional Enrichment Investigation of the DEGs in Both Two Groups

By the use of packages (ggplot2, enrichplot, and clusterProfiler), we performed GO and KEGG enrichment investigations based on DEGs [20]. Merely terms achieving *p* and *q* value < 0.05 had significant enrichment.

### 2.5. Investigation of Scores with Clinical Stages

The clinicopathological data of all CRC patients originated from TCGA. The assays were performed via the R language. According to the number of clinical stages for comparison, Kruskal-Wallis rank sum or Wilcoxon rank sum tests were used.

### 2.6. Univariate Cox Regression Investigations

With the use of the “survival” R package, we performed univariate Cox regression investigations for the identification of which DEGs were related to overall survival (OS). The DEGs with *p* < 0.05 were displayed with the use of forest plots.

### 2.7. Comprehensive Relationship Investigation in TICs

To screen the potential immune-related factors, in accordance with TCGA datasets, we applied the CIBERSORT algorithm for the examination of 22 infiltrating immunization cell types' proportions.

### 2.8. Cell Lines and Cell Transfection

A series of human CRC cell lines (Caco2, HCT116, SW620, SW480, and HT29) and human normal colon epithelial cell line (FHC) were purchased from the Cell Bank of Shanghai Institute of Biochemistry and Cell Biology (Shanghai, China). The cell lines were cultured in DMEM (iCell, Shanghai, China) supplemented with 10% FBS (GIBCO, Guangzhou, China) and 1% complex of penicillin and streptomycin in a 5% CO_2_ incubator at 37°C. The NAV3 siRNAs (si1 and si2) were purchased from JiMa Biological Corporation (Suzhou, Jiangsu, China). In accordance with the kits' protocols, the cell transfection was conducted using Lipofectamine 2000 reagent kits.

### 2.9. RNA Extraction and qRT-PCR Analysis

Total RNA was extracted from CRC cells by the use of a total RNA extraction kit (Vazyme, Nanjing, China). Total RNA was reverse transcribed into cDNA using a Reverse Transcriptase Kit (Beyotime Institute of Biotechnology). The expressions of NAV3 were examined via the ABI 7900HT Real-Time PCR System (Applied Biosystems, USA), using SYBR Green assays (TaKaRa, China), and GAPDH was used as the internal control. The primer sequences for NAV3 were designed and synthesized by Vazyme. The involved primers were as follows: NAV3 forward: AGCCTGTGCATACTGCTCTTC and NAV3 reverse: TGATTTTAACGCAAGCTGACAAG and GAPDH forward: GGAGCGAGATCCCTCCAAAAT and GAPDH reverse: GGCTGTTGTCATACTTCTCATGG.

### 2.10. Cell Proliferation Assays

Based on the manufacturer's instructions, CCK-8 was applied to examine cell proliferation. For the CCK-8 assays, 2 × 10^4^ cells/well were seeded in a 96-well plate for 24 h. Ten microliters of the Cell Counting Kit solution were added into each well at 0, 24, 48, and 72 hours after transfection. A microplate reader was applied to measure the absorbencies at each time point at 450 nm.

### 2.11. Statistical Analysis

We used R software for statistical investigations. With the use of the R packages “ggplot2” and “pheatmap,” respectively, the volcano plot and the heat map were obtained. The *t*-test was applied to test the differential expression of genes in cancer tissues compared to adjacent nonmalignant tissues. With the use of Kaplan-Meier estimates, the curves relating to survival were drawn; with the use of the log-rank test, we examined the difference between groups. For obtaining the hazard ratios (HRs) and 95% confidence intervals (95% Cis) of covariates in the investigations of overall survival, we adopted the Cox proportional hazard model. Only terms with both *p* and *q* value of < 0.05 were considered significantly enriched.

## 3. Results

### 3.1. The Prognosis Value of Scores in CRC Patients

**Figure 1:**
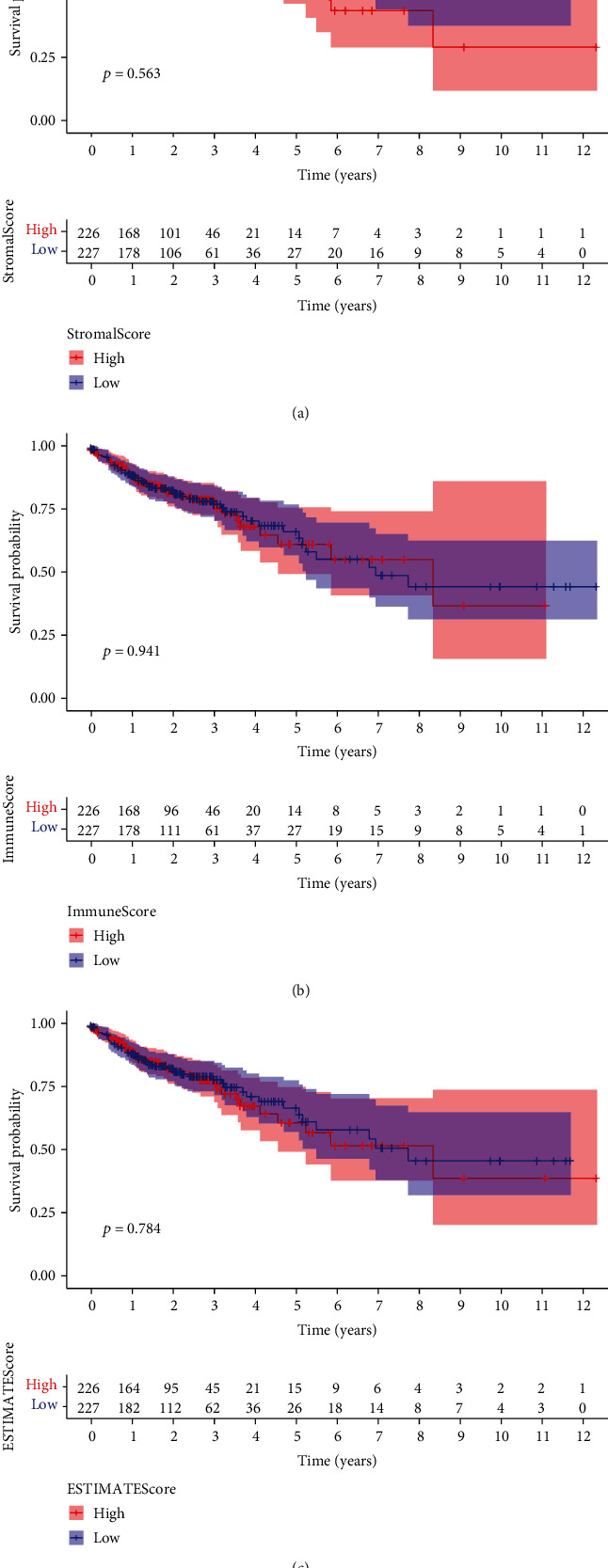
Relationships of ESTIMATEScore, StromalScore, and ImmuneScore with survival in CRC patients. Kaplan-Meier survival investigations of CRC patients suffering from low and high (a) ImmuneScores, (b) StromalScores, and (c) ESTIMATEScores.

To study the prognosis value of immune and stromal cell's evaluated proportion within CRC patients, we performed Kaplan-Meier survival investigation. The ESTIMATEScore referred to the sum of ImmuneScore and StromalScore denoting the comprehensive rate of both components within TME. According to the result, ImmuneScore (Figure 1(a)), StromalScore (Figure 1(b)), and ESTIMATEScore (Figure 1(c)) were insignificantly related to the total survival. However, we can observe a possible trend of higher ImmuneScore displaying a poor prognosis in CRC patients. More samples are needed to further determine the clinical significance of scores within patients suffering from CRC.

### 3.2. Relationship of the Scores with Clinical Characteristics in CRC Patients

**Figure 2:**
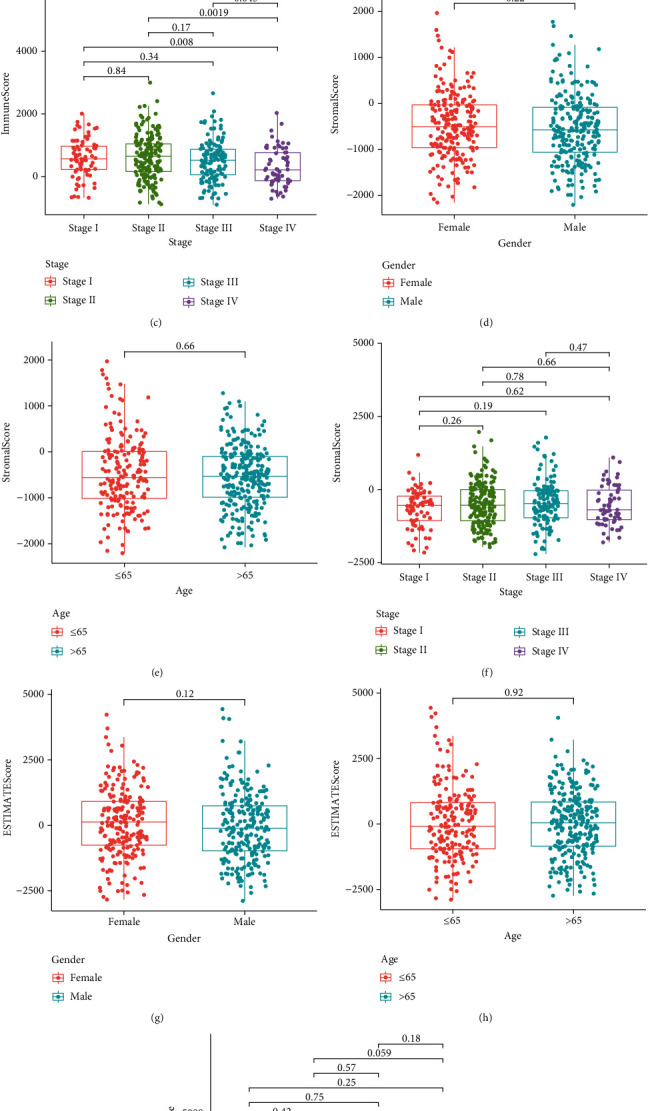
Relationship of scores showing clinicopathological staging features: (a–c) ImmuneScore in gender, age, and stage; (d–f) StromalScores in gender, age, and stage; (g–i) ESTIMATEScores in gender, age, and stage.

Subsequently, the relationship of the proportion of immune and stromal cells and patients' clinical characteristics including gender, age, and stage of tumor was investigated. The results showed that there is no distinct relationship between ImmuneScore and gender and age (Figures 2(a) and 2(b)). However, CRC specimens with an advanced tumor stage showed a decreased ImmuneScore (Figure 2(c)). On the other hand, we did not observe a distinct relationship of StromalScore (Figures 2(d)–2(f)) and ESTIMATEScore (Figures 2(g)–2(i)) with patients' clinical features.

### 3.3. DEGs Related to Tumor Microenvironment

**Figure 3:**
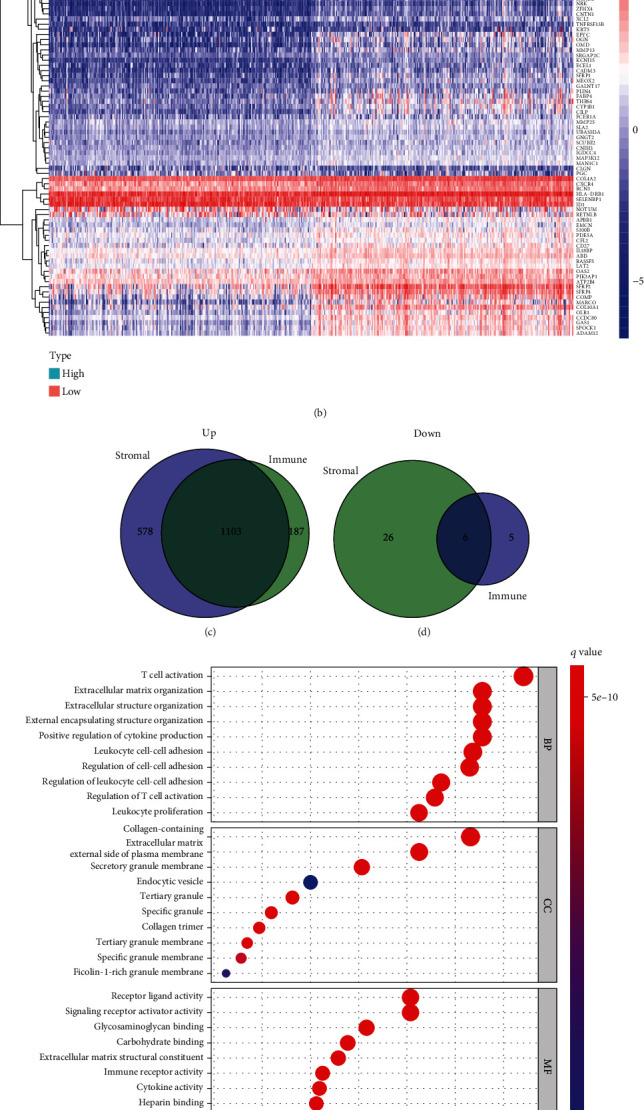
DEGs shared by the StromalScore and ImmuneScore and functional enrichment investigation. (a) The heat map of DEGs obtained in accordance with the ImmuneScore. (b) The heat map of DEGs obtained in accordance with the StromalScore. (c, d) Venn plots showing common increased and reduced DEGs shared by the ImmuneScore and StromalScore. (e, f) GO and KEGG assays with the use of the above DEGs.

The comparison assays between low- and high-score specimens were performed. We obtained 1322 ImmuneScore-associated DEGs and 1692 StromalScore-associated DEGs and exhibited the 100 most distinctly downregulated and upregulated genes (Figures 3(a) and 3(b)). Then, we picked 1109 TME-associated DEGs, of which 1103 were upregulated genes and six were downregulated genes (Figures 3(c) and 3(d)). GO enrichment investigation of DEGs was performed. It showed that DEGs were mainly distributed in T cell activation, extracellular matrix organization, collagen-containing, secretory granule membrane, and receptor ligand activity (Figure 3(e)). According to KEGG assays, DEGs had significant roles in cytokine-cytokine receptor interaction, chemokine signaling pathway, cell adhesion molecules, and Th17 cell differentiation (Figure 3(f)).

### 3.4. The Identification of Survival-Related and TME-Related Genes

**Figure 4:**
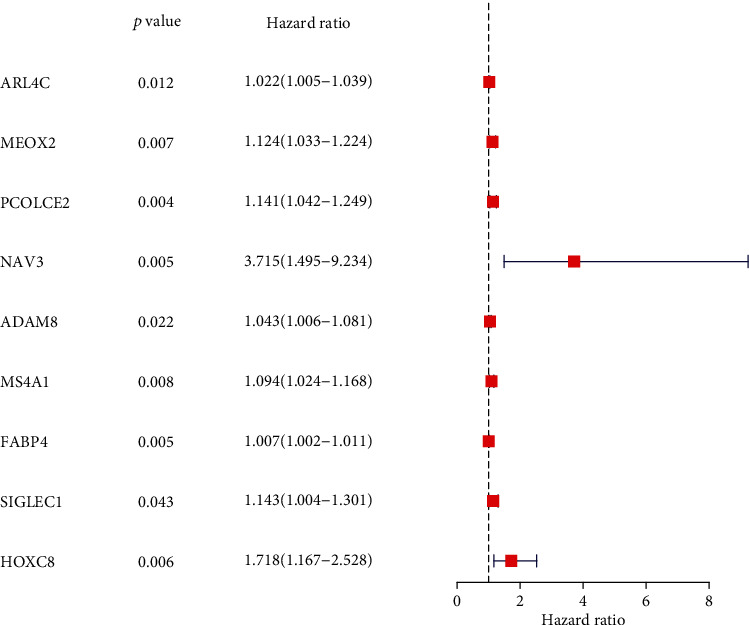
Univariate Cox regression investigation of DEGs.

For screening the survival-related genes in CRC, we performed univariate Cox regression with the use of the above 1109 TME-related DEGs. According to Figure 4, we identified nine survival-related genes, including ARL4C, MEOX2, PCOLCE2, NAV3, ADAM8, MS4A1, FABP4, SIGLEC1, and HOXC8. Given that NAV3 showed the relatively higher *p* value and HR value, we chose it for further study.

### 3.5. NAV3 Expression in CRC and Its Clinical Significance

**Figure 5:**
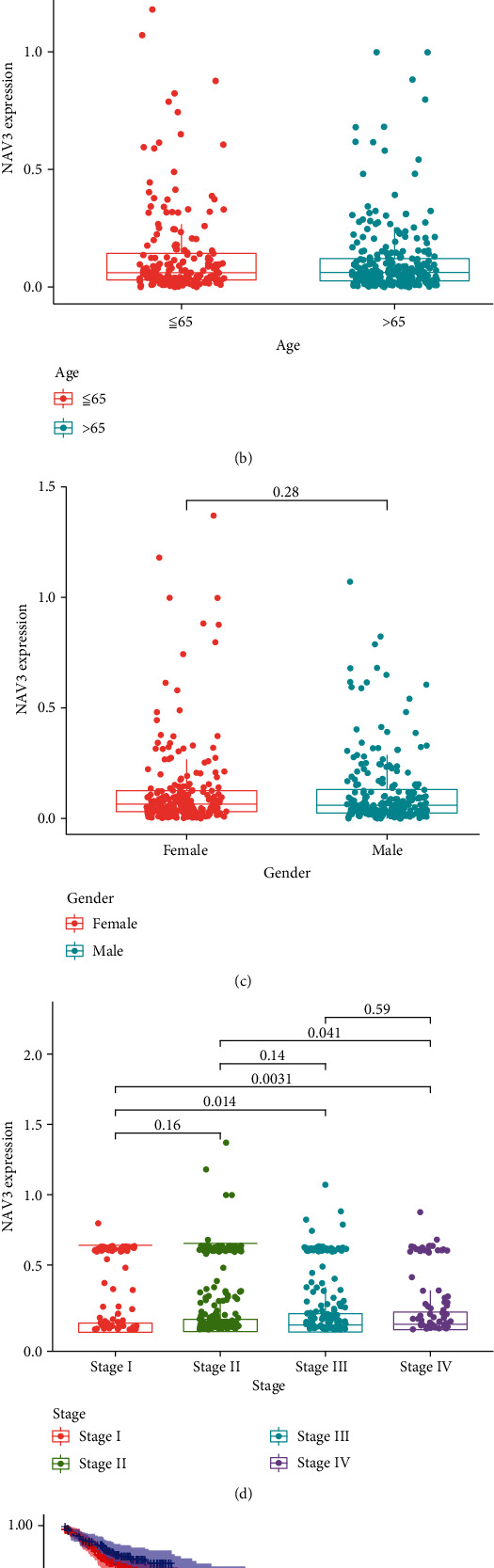
The expression of NAV3 and its clinical significance in CRC. (a) The expression of NAV3 was distinctly downregulated in CRC specimens. (b, c) The expressions of NAV3 in groups which were divided by age and gender. (d) Higher levels of NAV3 were observed in CRC specimens at different stages. (e) Kaplan-Meier curves estimating the overall survival according to the expression of NAV3 in patients with CRC.

**Figure 6:**
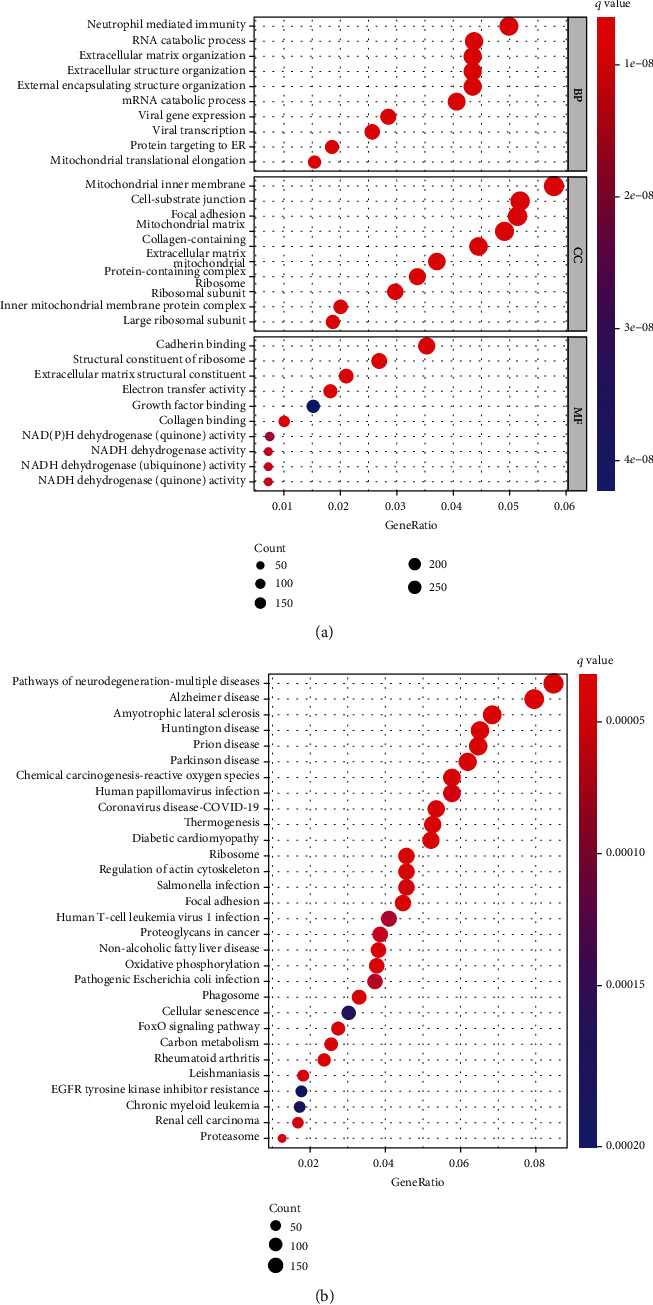
Pathway enrichment investigations of NAV3-related DEGs. (a) The numbers of genes enriched in each GO category. (b) KEGG pathways are analyzed, and the top 30 pathways are mapped.

As revealed by the analysis of TCGA, NAV3 expression was distinctly downregulated in CRC specimens in contrast to nontumor specimens (Figure 5(a)). Then, we analyzed the clinical relationship of NAV3 expression and found that there was no distinct relationship between NAV3 expression and age and gender (Figures 5(b) and 5(c)). Importantly, NAV3 expression noticeably increased within CRC specimens with stage IV and III than stage I (Figure 5(d)). Moreover, as revealed by the results of Kaplan-Meier methods, patients containing great NAV3 expression had a lower total survival in contrast to patients achieving low NAV3 expression (Figure 5(e)). For the exploration of the potential function of NAV3 in CRC, we performed GO and KEGG with the use of the dysregulated genes related to NAV3 expression in CRC. According to Figure 6(a), as revealed by GO enrichment investigation result, DEGs were mainly distributed in neutrophil-mediated immunity, RNA catabolic process, mitochondrial inner membrane, cell-substrate junction, cadherin binding, and structural constituent of ribosome. Moreover, as revealed by KEGG enrichment investigation, DEGs had major roles in Alzheimer disease, amyotrophic lateral sclerosis, Huntington disease, FoxO signaling pathway, and human papillomavirus infection (Figure 6(b)).

### 3.6. Patterns of Tumor-Infiltrating Immune Cells Related to NAV3 Expression

**Figure 7:**
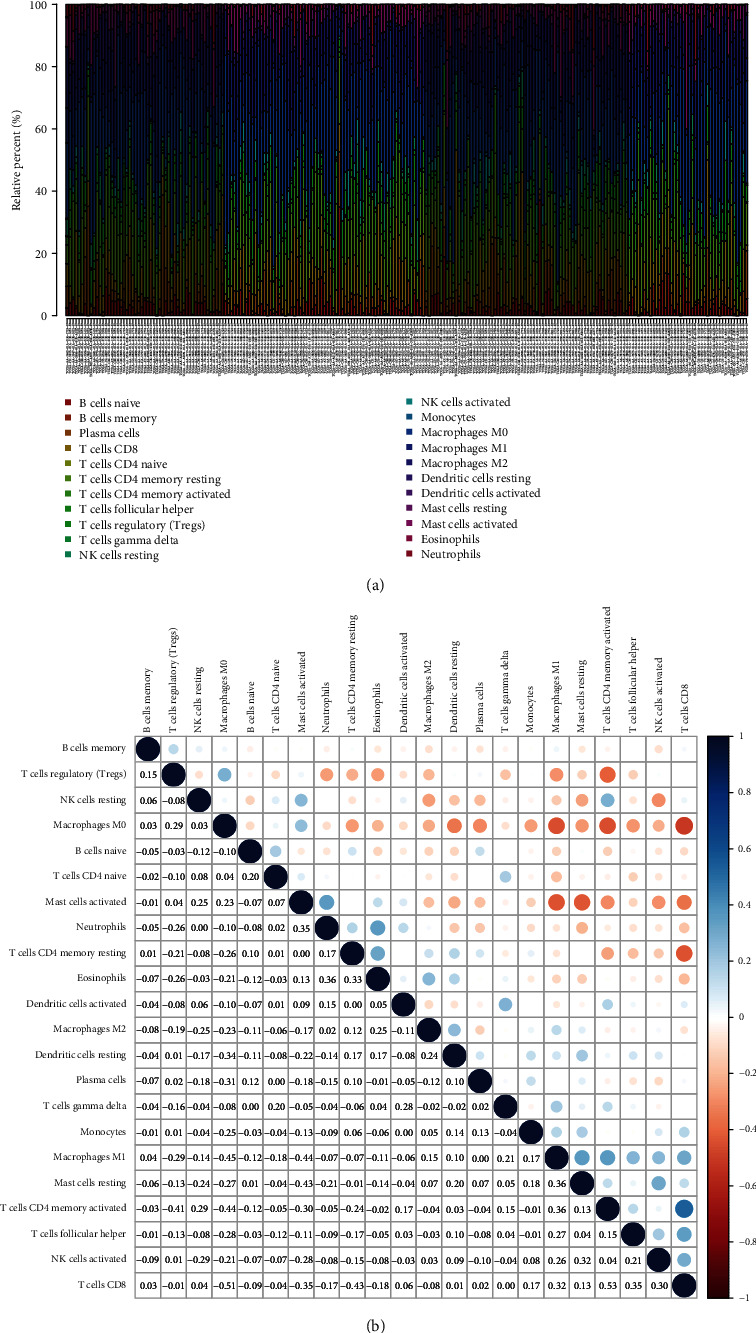
TIC profile within tumor samples and relationship investigation. (a) The landscape of immune infiltration in CRC tissues. (b) Relationship matrix of all 22 TIIC proportions.

**Figure 8:**
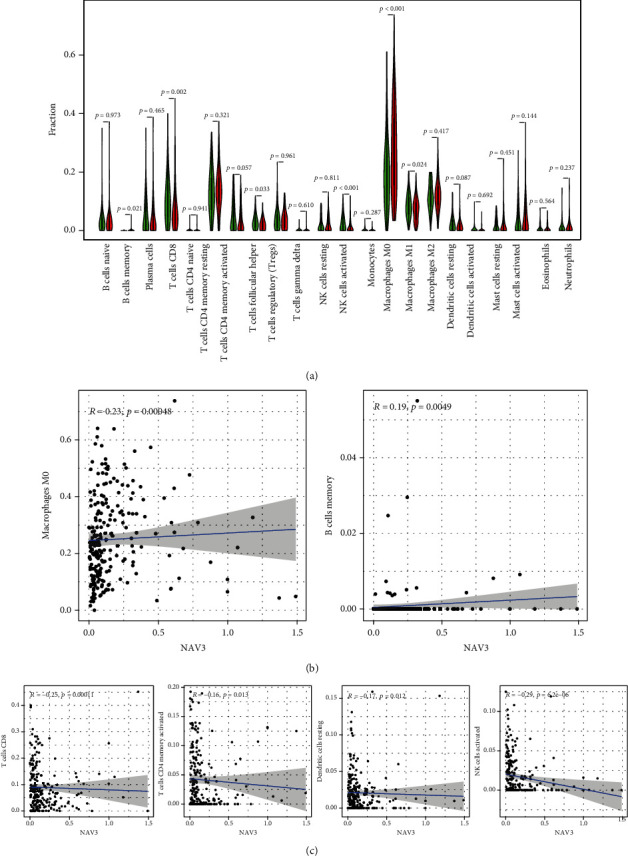
Relationship of TIC proportion with NAV3 expression. (a) The levels of 21 types of immunization cells in the two groups (high and low). (b, c) The relationship investigation between the levels of 21 types of immunization cells and the levels of NAV3 in CRC.

Whether NAV3 expression had a relationship to immune cell infiltration within CRC was explored. We qualified CRC samples within TCGA cohort with *p* < 0.05 based on the use of the CIBERSOFT algorithm. We summarized the immune infiltration landscape within CRC acquired in the tumor sample under the arrangement of low to high NAV3 expression (Figures 7(a) and 7(b)). As revealed by the result of the difference and relationship investigations, six types of TICs had correlations with NAV3 expression. To be specific, two types of TICs had a positive relationship to NAV3 expression (e.g., macrophage M0 and B cells memory) (Figures 8(a) and 8(b)). In addition, four TIC types had negative correlations with NAV3 expression, including NK cells activated, dendritic cells resting, T cells CD4 memory activated, and T cells CD8 (Figure 8(c)).

### 3.7. Knockdown of NAV3 Suppressed the Proliferation of CRC Cells

**Figure 9:**
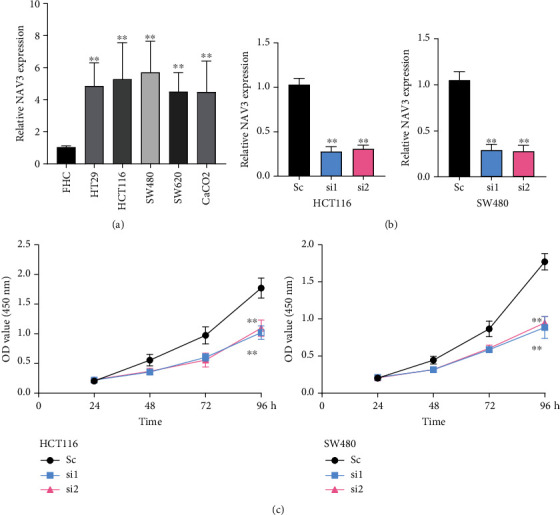
NAV3 promoted CRC cell proliferation in vitro. (a) NAV3 expressions in five CRC cells examined via RT-qPCR. (b) The efficiency of NAV3 silencing was examined through RT-qPCR in the HCT116 and SW480 after the transfection with siRNAs. (c) CCK-8 experiments confirming that silencing of NAV3 inhibited the proliferation of HCT116 and SW480 cells. ^∗∗^*p* < 0.01.

To confirm the levels of NAV3 in CRC cells, we performed RT-PCR to examine the expressions of NAV3 in several CRC cell lines. As shown in Figure 9(a), we found that NAV3 expressions were distinctly increased in five CRC cells compared with FHC cells. In addition, HCT116 and SW480 cells were transfected with the NAV3 siRNAs. Their effects were demonstrated through RT-PCR (Figure 9(b)). CCK-8 assays confirmed that silencing of NAV3 distinctly promoted the proliferation of the HCT116 and SW480 cells (Figure 9(c)). Our findings further indicated NAV3 as an oncogene in CRC.

## 4. Discussion

In the last twenty years, growing studies highlighted the important roles of TME in tumor progression [21]. Several types of TICs possessing tumor-promotive or tumor-suppressive functions infiltrate the TME [22, 23]. Therefore, the discovery of novel biomarkers influencing immunities of TME could point out novel anticancer channels. In this study, immune component proportion within TME might be related to the clinical outcome of CRC. Our data may provide a novel marker for CRC patients.

Growing evidences have confirmed the anticancer abilities of immunization cells due to their functions in inducing anergy-associated genes in T cells, thus resulting in the suppression of the anticancer characteristics. As an immunosuppressive receptor, PD-1 was found to exhibit a suppressive role during the effective stages of T cell activation via modulating suppressor pathways [24, 25]. An immunotherapy strategy is likely to be expanded by gaining insights into tumor immune escape mechanisms and the TME. The development of CRC immunotherapy achieves great progresses, and numerous immune checkpoint inhibitors (ICIs) were used clinically and led to encouraging results. Yet some patients still responded poorly to treatments. Furthermore, clinical practices reported adverse events and side effects, probably limiting the use of similar treatments [26, 27]. Based on the analysis of CRC transcriptomic data from TCGA, we found several survival-related genes in CRC. Importantly, we focused on NAV3 which exhibited a most significant role in predicting the prognosis of CRC patients. In addition, KCNN4 expression had a correlation with the clinical stage, thus confirming its relationship to poor patient prognosis. Our data highlighted NAV3 as a potential prognosis marker and therapeutic target in CRC.

NAV3 was a tumor suppressor gene which was expressed in both the central and peripheral nervous systems. In recent years, several studies have reported that NAV3 expression was distinctly downregulated in several types of tumors, such as glioma, breast cancer, and uterine leiomyoma [15, 28, 29]. Notably, low NAV3 expression was correlated with poor prognosis in these cancers. Importantly, the previous study by Uboveja et al. reported that knockdown of NAV3 and p73 expression distinctly promoted the migration and invasion rate of CRC cells. Also, NAV3 knockdown downregulated E-cadherin expression and upregulated other prominent mesenchymal markers' expressions (e.g., fibronectin, vimentin, Snail, and N-cadherin). These findings suggested that NAV3 acts as a tumor suppressor in colon cancer [30]. However, the expression and clinical significance of NAV3 in CRC have not been investigated. In this study, we found that NAV3 expression was distinctly downregulated in CRC and predicted a favorable prognosis in CRC patients, which was not consistent with the findings of Uboveja et al. For the analysis of the correlation of NAV3 expression and the TME, we analyzed the relationship between NAV3 and a wide variety of TIC subtypes. The results showed that the expression of NAV3 was correlated with a variety of immunization cells, including B cells memory, dendritic cells resting, macrophages M0, NK cells activated, T cells CD4 memory activated, and T cells CD8. Our findings suggested that NAV3 may affect the immune status of the TME and CRC prognosis. Finally, we performed functional assays and confirmed that NAV3 knockdown distinctly suppressed the proliferation of CRC cells.

However, some limitations of this study should be noted. First, although the accumulated data of high-throughput investigation from a large number of samples have been optimally applied, further verification through prospective studies is necessary. Second, the biological mechanisms of NAV3 in CRC are still unknown. Accordingly, further genetic- and experiment-based studies with larger samples and experiment validation are required.

## 5. Conclusion

This study confirmed that NAV3 optimized the TME and was lowly expressed in CRC. Decreased expression of NAV3 had a relationship with downregulated levels of a wide variety of TICs and was an independent prognosis factor in CRC. For this reason, this study presented novel ideas of NAV3's promotive function in the TME and NAV3's potential to be a prognosis biological marker in CRC.

## Data Availability

The data used to support the findings of this study are available from the corresponding authors upon request.
